# Exploring the Effects of Different Types of Surfactants on Zebrafish Embryos and Larvae

**DOI:** 10.1038/srep10107

**Published:** 2015-06-08

**Authors:** Yanan Wang, Yuan Zhang, Xu Li, Mingzhu Sun, Zhuo Wei, Yu Wang, Aiai Gao, Dongyan Chen, Xin Zhao, Xizeng Feng

**Affiliations:** 1 State Key Laboratory of Medicinal Chemical Biology, College of Life Science, Nankai University, Tianjin 300071, China; 2 Tianjin Key Laboratory of Tumor Microenvironment and Neurovascular Regulation, Department of Histology and Embryology, School of Medicine, Nankai University, Tianjin 300071, China; 3 The Institute of Robotics and Automatic Information Systems, Nankai University, Tianjin 300071, China

## Abstract

Currently, surfactants are widely distributed in the environment. As organic pollutants, their toxicities have drawn extensive attention. In this study, the effects of anionic [sodium dodecyl sulphate (SDS) ], cationic [dodecyl dimethyl benzyl ammonium chloride (1227)] and non-ionic [fatty alcohol polyoxyethylene ether (AEO) ] surfactants on zebrafish larval behaviour were evaluated. Five behavioural parameters were recorded using a larval rest/wake assay, including rest total, number of rest bouts, rest bouts length, total activity and waking activity. The results revealed that 1227 and AEO at 1 μg/mL were toxic to larval locomotor activity and that SDS had no significant effects. Moreover, we tested the toxicities of the three surfactants in developing zebrafish embryos. AEO exposure resulted in smaller head size, smaller eye size and shorter body length relative to SDS and 1227. All three surfactants incurred concentration-dependent responses. Furthermore, *in situ* hybridisation indicated that smaller head size may be associated with a decreased expression of *krox20*. The altered expression of *ntl* demonstrated that the developmental retardation stemmed from inhibited cell migration and growth. These findings provide references for ecotoxicological assessments of different types of surfactants, and play a warning role in the application of surfactants.

Water pollution is a major threat to the global ecosystem. The sewage from domestic washing, agriculture, and industry endangers the aquatic ecosystem and public health, and water pollution has aroused considerable public attention around the world in recent decades^1^,^2^. Surfactants have been successfully utilised in wetting, emulsification, solubilisation, sterilisation and detergency due to their physicochemical characteristics^3^,^4^,^5^,^6^. The annual global production of surfactants was approximately 13 million metric tons in 2008^7^, and production is currently growing. These chemicals are widely considered safe at low concentrations^8^,^9^. However, Britton noted that this approach to commercial surfactants should not be extended to every scenario, such as the waste water from hospitals and laundries^10^. The environmental risks associated with any large volume of chemicals warrant study.

Studies have previously assessed the toxicity and safety of surfactants^11^,^12^,^13^,^14^,^15^. Masakorala *et al.* examined the toxicities of sodium dodecyl sulphate (SDS), Triton X-100 (TX) and hexadecyltrimethylammonium bromide (HDTMA) in marine macroalgae and found that all of them reduced the efficiency of photochemical energy conversion, and SDS had a lesser effect compared to TX and HDTMA^16^. Vaughan *et al.* tested the acute toxicity of anionic, cationic and non-ionic surfactants in zebrafish embryos and adults. Their study indicated that embryos are as sensitive to cationic and non-ionic surfactants as adult fish but may be more sensitive to anionic surfactants^17^. However, few studies have investigated the developmental impacts of surfactants on aquatic animals. Moreover, to the best of our knowledge, there have been few studies on the effects of surfactants on rest/wake behaviour. Zebrafish, as an animal model, present many advantages over other animals. Their high homology with the human genome^18^, short developmental cycle and high fecundity were the primary reasons for choosing zebrafish as experimental subjects. Zebrafish embryos can develop to larvae in 3 days post fertilisation (dpf) and to adult fish in 3 months post fertilisation. One adult male and one adult female zebrafish can produce approximately 200 fertilised eggs. Thus zebrafish have long been considered a well-suited animal model for ecotoxicological^19^,^20^, developmental^21^, and neuroscientific research^22^,^23^,^24^. Therefore, we chose zebrafish embryos and larvae as the platform to assess the toxicity of surfactants *in vivo*. Surfactants are usually categorised into anionic, cationic, or non-ionic compounds according to the charge of the hydrophilic group^25^. We selected three representative, commercially important surfactants for further environmental risk assessment^26^,^27^. SDS, as an anionic surfactant, is widely used in laundry, shampoos and pharmaceuticals^28^. SDS is also used for polyacrylamide gel electrophoresis to determine the molecular weight of proteins in biochemical experiments^29^. The concentrations of SDS were 1.14-3.92 μg/mL in river water samples collected from 12 stations of river in Turkey, 4-24 μg/mL in raw sewage of UK^30^. Dodecyl dimethyl benzyl ammonium chloride (1227) is a cationic surfactant used to sterilise and remove algae, and is also applied in circulating water cooling systems. The concentrations of benzalkonium chlorides with carbon chains 12 (BAC-C12) and 14 (BAC-14), the 1227 analogues, were 2.7-5.8 μg/L and 6.3-36.6 μg/L in five waste water treatment plants (WWTP) of USA^31^. Fatty alcohol polyoxyethylene ether (AEO-7) is a non-ionic surfactant that is primarily used in detergents and emulsifiers^32^. The concentrations of AEO in seawater and fresh water were below 50 μg/L^33^. Although the concentrations in the fresh water and seawater were rather low, the concentrations of surfactants from contaminated water were higher, including the waste water from hospitals, laundries and so on. In order to explore the possible effects of surfactants on organism at higher concentrations, we apply an automatic, high-throughput and rapid video tracking system to observe larval rest/wake behaviour for 48 hours and evaluate the behavioural impact of surfactants. Furthermore, we examined the effects of three surfactants on zebrafish embryonic development from 1.25 hours post fertilisation (hpf) to 120 hpf by morphological analysis and gene expression. The present article is, to our knowledge, the first concerning the toxicity of surfactants on larval rest/wake behaviour. The results indicated that the toxicity of AEO in rest/wake behaviour and embryos development is much more than that of SDS and 1227 at the concentration higher than environment-related concentrations, providing references for ecotoxicological assessments of different types of surfactants with widespread application, and playing a warning role in the application of surfactants.

## Results

### Rest/wake behavioural assay in larval zebrafish and characteristics of three surfactants

**Figure 1:**
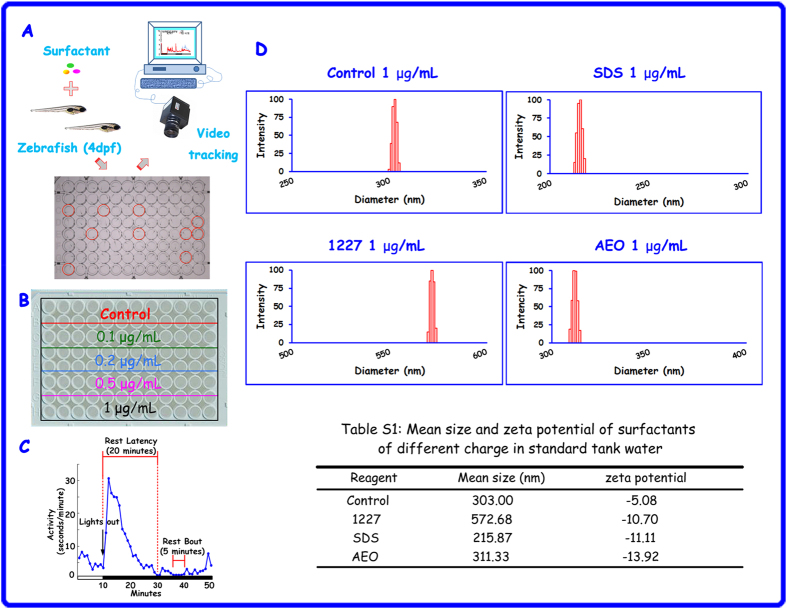
Locomotor activity assay in larval zebrafish and characteristics of surfactants. (**A**) The process for the rest/wake behavioural assay. The picture of the 96-well plate above is from the video tracking system. The well with larval movement is highlighted using a red circle. The duration of movement during each minute was recorded to obtain the raw data. (**B**) The concentrations were 0.1, 0.2, 0.5, and 1 μg/mL. Standard system water was used to establish controls. Sixteen larvae were tested in each group. (**C**) In the larval rest/wake behavioural assay, we defined five parameters, including total time spent at rest, the number of rest bouts, rest bout length, total activity and waking activity. The analysis of these five parameters is illustrated in the line graph. (**D**) The diameter distribution and zeta potential of SDS, 1227 and AEO in standard system water were determined using DLS. The control was also measured for comparison.

Our experimental protocols briefly referred to Schier’s method, in which an automatic video recording system was employed to observe the rest/wake behaviour of zebrafish larvae exposed to thousands of psychotropic drugs, followed by the assessment of the neural pathways affected by these compounds^34^. To obtain the locomotor activity data of larval rest/wake behaviour for 48 continuous hours, a custom program was developed and integrated the use of a camera (Fig. 1A). With anionic and non-ionic surfactants, chronic toxicity usually occurs at concentrations greater than 0.1 μg/mL^35^; thus, we used concentrations of 0.1, 0.2, 0.5, and 1 μg/mL to determine various concentration effects (Fig. 1B). The rest/wake behaviour of zebrafish is very complex. Therefore, to assess the diverse influences of anionic, cationic and non-ionic surfactants, five parameters were analysed in the larvae, including rest total, the number of rest bouts, rest bouts length, total activity and waking activity. These parameters represent different characteristics of sleep behaviour. Sleep time and sleep maintenance are shown in Fig. 1C. The dynamic light scattering (DLS) identification results showed that the particle sizes of SDS, 1227 and AEO were approximately 215.87 nm, 572.68 nm and 311.33 nm, respectively; the zeta potentials of SDS, 1227 and AEO dissolved in standard system water were electronegative. The critical micelle concentration (CMC) is an important physicochemical property of surfactants. When the concentration is above the CMC, surfactants will prone to form micelle. The tested concentrations in our study were below respective CMC, so they existed in the form of particle. (Fig. 1D).

### Clustering analyses for the behavioural phenotypes induced by SDS, 1227, and AEO

**Figure 2:**
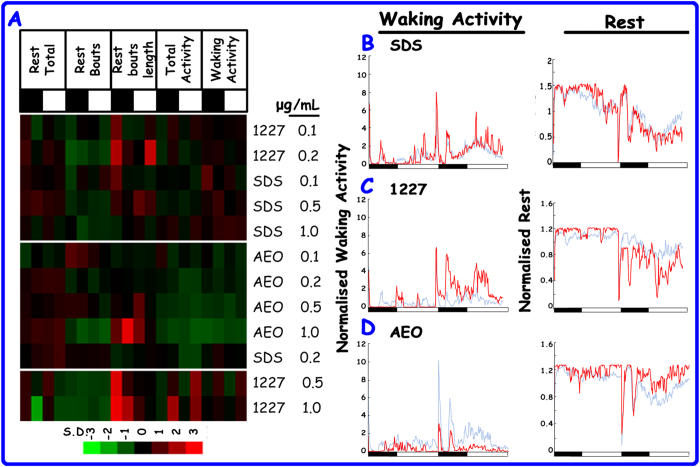
Behavioural similarities induced by SDS, 1227 and AEO. (**A**) In the k-means clustergram, each row represents a chemical at a unique dose, and each column represents a behavioural parameter. The black bars indicate the night measurements, and the white bars indicate the day measurements. From left to right, the parameters were: total time spent at rest, number of rest bouts, rest bout length, total activity and waking activity. These parameters were normalised as standard deviations from the control values. The red and green colours indicate that the values are higher and lower than the controls, respectively. (**B-D**) Total rest and waking activity are averaged in 10-minute intervals and then normalised to control values. The red and blue lines indicate the exposed and control groups, respectively. The black bars represent the night measurements, and the white bars represent the day measurements.

Based on the five normalised parameters, we performed k-means clustering analyses according to shared behaviours. K-means clustering was conducted with k values from 2 to 4; we chose k = 3 for the detailed analyses (Fig. 2A). The results with k = 2 and k = 4 are presented in Figure S1. We established four concentrations for SDS, 1227 and AEO, respectively. As shown in the clustergram, 0.1 0.2 μg/mL 1227 co-clustered with SDS 0.1, 0.5, and 1 μg/mL. AEO at the four doses was in a separate category. 1227 at high doses belonged to one class. These results demonstrate that the behavioural changes induced by 1227 and SDS were very similar to each other but differed from the behavioural profiles caused by AEO. In the right panel, waking activity and rest were normalised to the control values for SDS, 1227 and AEO at 1 μg/mL (Fig. 2B–D). Based on the time series, SDS did not significantly affect the two parameters. On the second day, 1227 increased waking activity and decreased rest. AEO selectively decreased waking activity and increased the total amount of time spent rest during the day. The time series of SDS, 1227 and AEO at each dose are presented in Figure S2.

### Qualitative and quantitative analyses of behavioural changes induced by SDS, 1227 and AEO

**Figure 3:**
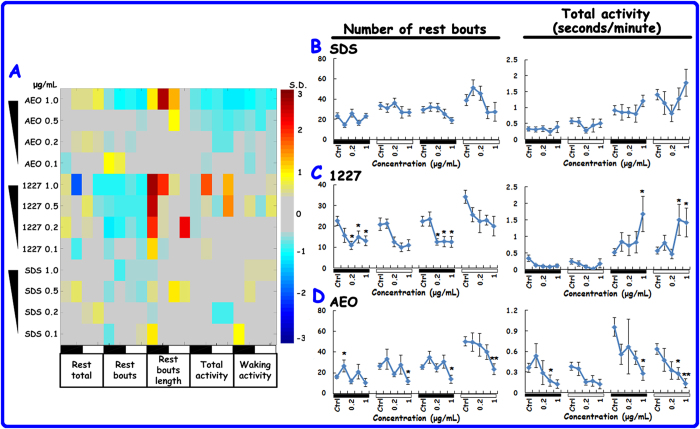
Qualitative and quantitative analyses of behavioural changes caused by SDS, 1227 and AEO. (**A**) In the fingerprint, five parameters were described. From left to right, the parameters were: total time spent at rest, number of rest bouts, rest bout length, total activity and waking activity. Each row represents a chemical at a unique dose, and each column represents a behavioural parameter. The measurements are normalised as standard deviations from the control values. The blue and red colours indicate that the values were lower and higher relative to the control values. The black bars indicate the night measurements, and the white bars indicate the day measurements. (**B-D**) In the line graphs for the number of rest bouts and the total activity, each value indicates the average of ~48 larvae. The error bar represents the standard error of the means (SEM). The statistical significances were set to P < 0.05 (*) and P < 0.01 (**). The black bars indicate the night measurements, and the white bars indicate the day measurements.

Five behavioural parameters were further quantitatively analysed to compare the effects of the three surfactants on larval rest/wake behaviour. The line graphs of rest total, rest bouts length and waking activity are presented in Figure S3. After exposure to SDS, the treated groups showed no significant changes relative to the controls (Fig. 3A,B, and S3). The 1227-treated group displayed more marked effects. The number of rest bouts was remarkably decreased at night in this group. The lowest observed effective concentration (LOEC) was 0.2 μg/mL. The animals’ total activity increased during the second day. The rest total, rest bouts length and waking activity were not significantly different from those of the controls (Fig. 3C and S3). AEO decreased the number of rest bouts and total activity at 0.5 and 1 μg/mL (Fig. 3D) and increased the rest total and rest bouts length at 1 μg/mL. Waking activity declined as the concentration increased (Figure S3). To compare the differences between these behavioural measures during the day vs those during the night, we calculated the total values for the number of rest bouts and the total day and night activity, respectively (Figure S4). 1227 and AEO caused significant changes at 0.2, 0.5, and 1 μg/mL, while SDS had little effect on the two parameters. From the SDS, 1227 and AEO analyses, we found that 1227 increased larval locomotor activity and AEO reduced this activity. In summary, 1227 and AEO were more toxic to the locomotor activity of zebrafish larvae than SDS.

### Toxicity of surfactants in developing zebrafish

**Figure 4:**
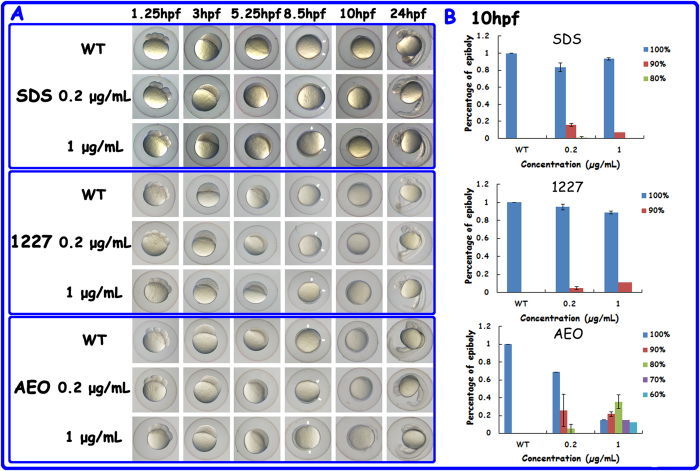
Effects of surfactant treatment on embryonic development. (**A**) Morphological characteristics of embryos treated with SDS, 1227 and AEO from 1.25 to 24 hpf. The white arrows represent the start and end of epiboly. (**B**) The epiboly percentages of the embryos exposed to the surfactants at 10 hpf. The error bar represents the standard error of the means (SEM). The statistical significances were set to P < 0.05 (*) and P < 0.01 (**). 0.2 μg/mL SDS (n = 131), 1 μg/mL SDS (n = 142); 0.2 μg/mL 1227 (n = 121), 1 μg/mL 1227 (n = 145); 0.2 μg/mL AEO (n = 140), 1 μg/mL AEO (n = 169).

To assess the toxicity of surfactants in zebrafish development, the fertilised eggs were exposed to surfactants and the effects on embryonic development were observed from 1.25 to 120 hpf. No morphological alterations were found between the control group and the treated groups from 1.25 to 5.25 hpf. At 5.25 hpf, a majority of embryos in each group had reached 50% epiboly. As development continued, the morphological characteristics of the zebrafish embryos at 8.5 and 10 hpf showed that the embryos exposed to surfactants displayed developmental delay compared with the controls and the statistical analyses showed that epiboly delay was the most pronounced in the AEO-treated groups (Fig. 4A,B and S5).

Developmental retardation increased as surfactant concentrations increased in all surfactant groups at 8.5 hpf. At 10 hpf, a majority of the embryos exposed to SDS and 1227 reached 100% epiboly, and the developmental delay in these embryos was less than in those treated with AEO. In accordance with the results observed at 8.5 hpf, however, the developmental retardation caused by each surfactant followed a concentration-dependent pattern at 10 hpf.

To further explore the toxicities of the three surfactants to embryonic development, the eye area, head area and body length of each embryo were measured at 28, 48, 72, 96, and 120 hpf (Figure S6). At 28 hpf, exposure to 1 μg/mL AEO caused smaller eyes and shorter body lengths. Smaller eyes, smaller heads and shorter bodies remained apparent at 48 hpf. The effects of AEO on larval embryonic development were reduced at 72 hpf and were not significant at 96 or 120 hpf. Although slight effects on eye sizes, head sizes and body lengths were also found in the SDS- and 1227-treated groups, there were no significant differences relative to those of the wild type from 28 to 120 hpf.

Mortality was also recorded to evaluate the toxicities of the three types of surfactants. No embryos died prior to 5.25 hpf. At 8.5 hpf, a sharp increase in mortality (35%) was observed in the 1 μg/mL AEO-treated group, whereas no significant differences were found in the groups treated with SDS or 1227. At 10 hpf, the mortality of the 1 μg/mL AEO-treated group increased to 53%, while the mortalities of the SDS- and 1227-treated groups remained under 10% (Figure S7).

### Gene expression of embryos exposed to surfactants

**Figure 5:**
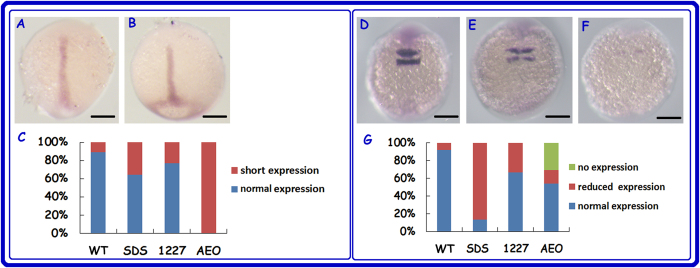
Expression of genes in controls and the surfactant-exposed embryos at 1  μg/mL. (**A**) Expression of *ntl* at 100% epiboly. (**B**) Reduced expression of *ntl* in the axial chordal mesoderm. (**C**) Statistical analyses of two types of *ntl* expression at 10 hpf in the controls and treated embryos. (**D**) Expression of *krox20* at 12 hpf. (**E**) Reduced expression of *krox20* in the treated embryos. (**F**) No expression of *krox20* in the treated embryos. (**G**) Statistical analyses of three types of *krox20* expression at 12 hpf in the controls and treated embryos. Scale bars = 200 μm.

Morphological defects are usually related to altered gene expression. Thus, we tested the expression patterns of *ntl* (no tail) and *krox20* using whole-mount *in situ* hybridisation. Epiboly involves the migration of many cell types^36^. To determine whether the delayed epiboly was related to a disorder of cell migration or to slow growth, we examined the expression of *ntl* in embryos at 10 hpf. *ntl* is a marker of the induction of the mesoderm, which gives rise to posterior body structures. This gene was expressed in the margin (5.25 hpf), axial chordal mesoderm, and notochord (10 hpf) during embryonic development^37^,^38^. As shown in Fig. 5A-C, *ntl* was expressed in the notochord in 90% of the embryos of the untreated group at 10 hpf, while 36% of the embryos in the 1 μg/mL SDS-treated group and 23% of the embryos in the 1 μg/mL 1227-treated groups exhibited short *ntl* expression in the axial chordal mesoderm and obvious expression in margin, which were silimar to the expression pattern of *ntl* at stage before 80% epiboly. In the 1 μg/mL AEO-treated group, all of the embryos displayed short *ntl* expression in the axial chordal mesoderm *Krox20* (also known as *Egr2*) is a transcription factor involved in vertebrate hindbrain segmentation, especially in the formation and specification of rhombomeres (r) 3 and 5, and expresses in r3 and r5^39^,^40^. At 12 hpf, most embryos expressed this gene normally in the control group, while the levels of *krox20* expression were lower in the surfactant-treated embryos. 25% of the embryos in the 1 μg/mL 1227-treated group and approximately 80% of the embryos in the 1 μg/mL SDS-treated group showed mildly reduced *krox20* expression. In the 1 μg/mL AEO-treated group, 15% of the embryos exhibited a mildly reduced *krox20* expression; 30% of these embryos showed no *krox20* expression (Fig. 5D-G).

In addition, qRT-PCR was performed to detect the expression of *ntl* and *krox20*. The result showed that the expression of *ntl* in surfactant treated groups was higher than that of control. While the expression of *krox20* in surfactant treated groups became lower. The extent of downregulation in AEO-treated group was the most obvious (Figure S8).

## Discussion

Because of the universal utilisation in emulsification, sterilisation and detergency, and large consumption, surfactants are widely discharged in environmental water, which maybe cause serious pollution and pose a threat to human health. Generally speaking, the surfactants in the aquatic environment can undergo rapid degraded by microorganism^41^,^42^. However, if the surfactants enter into anaerobic conditions, surfactants will accumulate in aquatic organisms and induce toxicity^43^,^44^. In this study we access the toxicity of three types of surfactants by examining their effects on behavioural activity and early development of zebrafish. We found that 1227- and AEO-exposure could significantly affect larval sleep patterns in a concentration-dependent manner. 1227 increased but AEO decreased larval locomotor activity. No obvious effection was detected in SDS-treated larval by behavioural activity. This behavioural analysis suggested that 1227 and AEO were more toxic to zebrafish larvae than SDS. Furthermore, developmental toxicity was caused in the embryos by SDS, 1227 and AEO treatment. We found that all three types of surfactants induced embryonic developmental retardation and the effect also followed a concentration-dependent pattern. The AEO-induced epibolic delay was the most pronounced, which was confirmed by the concomitant misexpression of *ntl*, a marker of epiboly. Smaller eyes, smaller heads and shorter body lengths were also observed in all three of the surfactant-exposed groups; however, the defects caused by AEO were the most severe, as indicated by the eye area, head area and body length as well as *krox20* expression.

In our research, the concentrations of surfactants used were 0.1, 0.2, 0.5 and 1 μg/mL. The maximum concentration approximated the environmental concentration of SDS^30^. Since SDS at highest concentration used in our study caused no obvious effects on zebrafish behaviour and early development, SDS in river water hardly exert an influence on the behavioural activity and early development of zebrafish. It has been reported that the concentrations of AEO in seawater and fresh water were below 50 μg/L, which was far below the concentration used in our study. However, total concentrations of AEs in influent wastewater (USA) ranged from 685-3,677 μg/L with an average concentration of 2569 μg/L^45^. We are not sure whether AEs make the influence like AEO, but as a precaution, we should decrease discharge of the waste containing AEs. No data about the 1227 in the environment were found, but the analogues BAC-C12 and BAC-C14 were detected 2.7-5.8 μg/L and 6.3-36.6 μg/L in five WWTP (USA), which were also far below the concentration in our study. So the toxicity of AEO and 1227 in environment can be omitted at lower concentrations.

Although the concentrations in the fresh water or seawater were rather low, the concentrations of surfactants from contaminated water were higher, including waste water from hospital, laundries and so on. It’s necessary to access the toxicity of surfactants at higher concentration, which would provide references for ecotoxicological assessments of different types of surfactants with widespread application, and play a warning role in the application of surfactants.

## Methods

### Zebrafish

The zebrafish (AB strain) were raised in a standard water system (KCl 0.05 g/L, NaHCO_3_ 0.025 g/L, NaCl 3.5 g/L, and CaCl_2_ 0.1 g/L, with 1 μg/mL methylene blue, pH 7.0) at a constant 28.5 °C with a 14 h light/10 h dark cycle. The zebrafish were fed frozen brine shrimp twice daily. The night prior to the start of a test, one male and one female zebrafish were transferred to a breeding tank. The next morning, sufficient eggs were collected from the breeding tanks and cultured in system water at 28.5 °C. The eggs were washed to remove the unfertilised eggs and debris. The embryos were stored with sufficient room to develop normally. The solutions were renewed three times per day. The larvae were used for behavioural assays at 4 dpf. All experimental protocols and procedures involving zebrafish were approved by the Committee for Animal Experimentation of the College of Life Sciences at Nankai University (no. 2008) and were performed in accordance with the NIH Guide for the Care and Use of Laboratory Animals (no. 8023, revised in 1996).

### Chemicals

SDS (99.5%) and 1227 (99.5%) were obtained from Aladdin Industrial Co., Ltd (Shanghai, China). AEO (99.5%) was purchased from Ai Keda Chemical Technology Co., Ltd (Chengdu, China). All surfactants were used as they were received without any further purification. All surfactant solutions were prepared freshly just prior to the experiments with standard system water to obtain stock solutions of 36 mg/mL, which were used to prepare the 0.1, 0.2, 0.5, and 1 μg/mL dilutions. To determine the diameter distribution and zeta-potential of the three surfactants in standard system water, we conducted the DLS.

### Larval sleep/wake assay for testing the behavioural activity of zebrafish treated with SDS, 1227 and AEO

First, to observe the concentration-toxicity relationships between three surfactants and zebrafish larvae, four doses (0.1, 0.2, 0.5 and 1 μg/mL) were used. Sixteen fish were tested for each group. After exposing the zebrafish larvae to the surfactants for 12 hours, we used a custom video tracking system to record their locomotor activity over 48 continuous hours. In this system, the larvae were pipetted into a 96-well plate. To minimise evaporation, the 96-well plate was sealed with a layer of plastic film and then put on an acrylic shelf. An infrared LED array was located below the shelf as a backlight source. An acrylic diffuser was placed above the infrared LED array. A camera (MV-VS078FM, MicroVision, Japan) with a fixed-angel megapixel lens (MP5018, Computer) was fixed on a carne for vertical observations. The camera can yield high-quality images with a resolution of 1024*768 at 10 fps. A program was developed with Microsoft Visual Studio 2005 to record the larval rest/wake behaviour. The algorithm was based on the subtraction of the adjacent frames. The system was maintained in a lab at a constant 28.5 °C with illumination from 7:00 AM to 9:00 PM. A 48-hour continuous time series of the larval rest/wake behaviour was stored for offline analyses.

### Data analyses

We obtained a time series from the raw data of the offline file. Each number in the time series represented the number of movements by one larva in one minute during the 48 consecutive hours. To assess the larval rest/wake behavioural changes, five parameters were extracted from the raw data: rest total, the number of rest bouts, rest bouts length, total activity and waking activity. These parameters were calculated for each experimental day and night. Any minute with less than 0.3 seconds of total movement was defined as one rest minute. A rest bout was defined as a continuous series of rest minutes. The rest total was defined as the total minutes of rest during all experimental days and nights. The rest bout length was defined as the average length of a continuous string of rest minutes. The total activity was defined as the average amount of activity per animal. Waking activity was also defined as the average amount of activity after excluding all rest minutes.

We replicated the rest/wake behavioural assay three times for each surfactant on independent occasions. The value for each parameter is the average of all of the repeated experiments and is expressed as the means ± standard error of the mean (SEM). One-way ANOVA was conducted to assess differences between the four treated groups and the control group. P-values <0.05 were considered to indicate significance. Among the five parameters, total rest and waking activity were normalised to the controls and averaged in 10-minute intervals; these were then used to generate a time series. The controls were also plotted for comparison. For each surfactant, five parameters were tested at four doses, which were normalised to the controls and constituted a feature vector. These feature vectors were used to preform k-means clustering analyses based on the Euclidean distance. The feature vectors were taken as behavioural fingerprints for each surfactant.

### Effects of SDS, 1227 and AEO on early zebrafish development

The fertilised eggs were collected immediately after natural spawning. Embryos were selected at the 1-cell stage and exposed to one of three surfactants at concentrations of 0, 0.2, and 1 μg/mL, respectively. All embryos were observed under a stereomicroscope (Olympus ZX-10, Japan) at different stages from 1.25 to 120 hpf and photographed for morphological analyses. The embryos from 28 to 120 hpf were dechorionated using forceps and anesthetised via immersion in tricaine for photography under a stereomicroscope equipped with a digital camera (Panasonic). The head and eye areas and the body length of the embryos were measured using the Olympus BP2-BSW software. The exposure experiments were repeated four times, and the value of each parameter (percentage of epiboly, mortality, head area, eye area and body length) was expressed as the means ± standard error of the mean (SEM). One-way ANOVA was conducted to assess differences between the treated groups and the control group. P-values <0.05 were considered to indicate significance.

### Whole-mount *in situ* hybridisation

The embryos of each group were collected at 10 hpf (for *ntl*) and at 12 hpf (for *krox20*) and fixed in 4% paraformaldehyde pH 7.0 in phosphate-buffered saline overnight at 4 °C. Plasmids containing *ntl* and *krox20* were obtained from Professor Hongwei Zhang (Shandong University). Antisense probes and whole-mount *in situ* hybridisation were performed as previously described^46^.

### Quantitative RT-PCR

Total RNA was extracted from wild type and surfactants treated embryos at 10 hpf and 12 hpf using Trizol according to the manufacturer’s protocol (Invitrogen, USA), and was reverse-ranscribed by MMLV reverse transcriptase (Promega, Madison, WI) using the oligo (dT) primers.

Quantitative RT-PCR was performed using the SYBR Green Labeling System (BioRad, Hercules, CA). Conditions qRT-PCR included a denaturing step at 95 °C for 2 min, 40 cycles of 95 °C for 30 sec, 62 °C for 30 sec, and 72 °C for 30 sec for real time plate read, and a final extension at 72 °C for 5 min. beta-actin was used for data normalization. Primer sequences included:

beta-actin, Forward 5′-TGTGGGCGTGGTGTTTGAGGTAC-3′,

Reverse 5′- CCGGATCTCGATGAGTCAGGGAA-3′;

ntl, Forward 5′-GTCAAACTCTCCAATAAACTCA-3′,

Reverse 5′-AGCGGTAATCTCTTCATTCT-3′;

krox20, Forward 5′-CATCCTACTCCTCTCCAAAG-3′,

Reverse 5′- TAGCGTGAAGTTCCTGATAG-3′.

### Software

One-way ANOVA was conducted using SPSS 19.0. We applied Cluster 3.0 for k-means clustering analyses and Java Tree View and Microsoft Visio 2010 for visualisation. The line graphs and bar charts were plotted using Microsoft Excel 2010. The time series, behavioural fingerprint, and box plot were visualised with MATLAB R2011b (Math Works). All of the figures were assembled using Photoshop 7.0.

## Additional Information

**How to cite this article**: Wang, Y. *et al.* Exploring the Effects of Different Types of Surfactants on Zebrafish Embryos and Larvae. *Sci. Rep.* 5, 10107; doi: 10.1038/srep10107 (2015).

## Supplementary Material

Supplementary Information
