# Supplementary material for: Exploring the Effects of Different Types of Surfactants on Zebrafish Embryos and Larvae
Source: Sci Rep. 2015 Jun 8;5:10107. doi: 10.1038/srep10107 (PMC4459078; doi:10.1038/srep10107)
Supplement: Supplementary Information [file srep10107-s1.doc]

***Supporting information***

**Exploring the Effects of Different Types of Surfactants on Zebrafish Embryos and Larvae**

Yanan Wang^1,‡^, Yuan Zhang^1,‡^, Xu Li^2,‡^, Mingzhu Sun^3^, Zhuo Wei^1^, Yu Wang^1^, Aiai Gao^2^, Dongyan Chen^2,*^, Xin Zhao^3,*^, Xizeng Feng^1,*^

**Table of Contents**

**Supplementary Figures**

**Figure S1.** K-means clustering analyses;

**Figure S2.** The time series for SDS, 1227 and AEO at each dose;

**Figure S3.** The [quantitative](app:ds:quantitative) analyses for five behavioral parameters;

**Figure S4.** Statistical analyses of number of rest bouts and total activity during both the day and night;

**Figure S5.** Morphological characteristics of embryos treated with surfactants from 1.25 to 24 hpf;

**Figure S6.** Early development of embryos treated with surfactants from 28 to 120 hpf;

**Figure S7.** Mortality of embryos treated with surfactants from 1.25 to 10 hpf.

**Figure S8.** The expressions of *krox20* (A) and *ntl* (B) by qRT-PCR in zebrafish larvae.

**Figure S1**

**K=2 K=3**

**
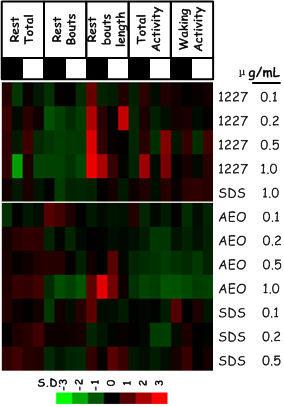

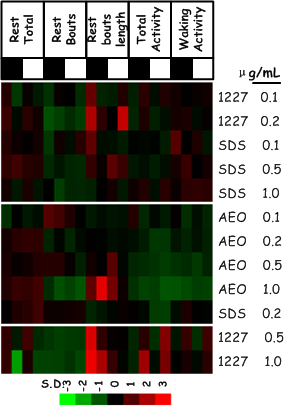
**

**K=4**

**
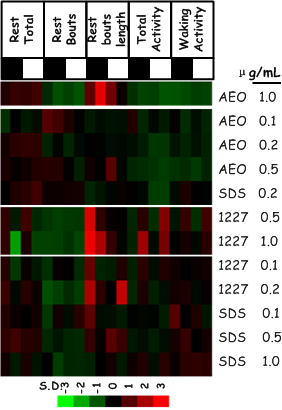
**

**Figure S1**. K-means clustering. In the k-means clustergram, each row represented a chemical at a dose, and each column represented a behavioral parameter. The black bars indicated night measurements, and the white bars indicated day measurements. From left to right, these parameters were: rest total, number of rest bouts, rest bouts length, total activity and waking activity. These parameters were normalised as standard deviations from the controls. The red and green colors indicated that the values are higher and lower than the controls, respectively.

**Figure S2** SDS


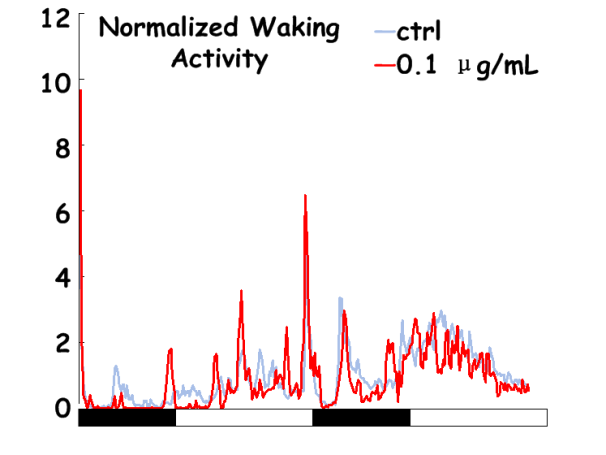

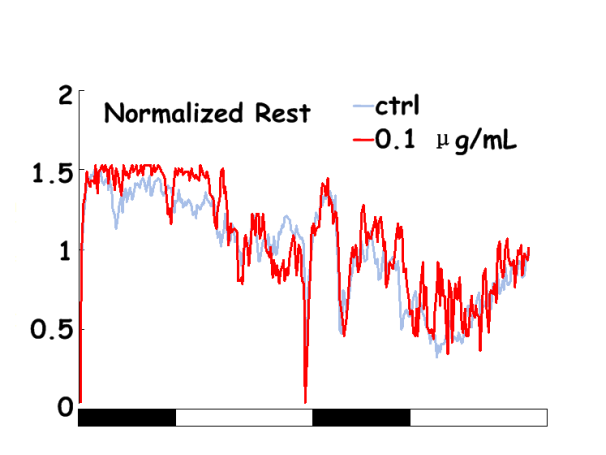


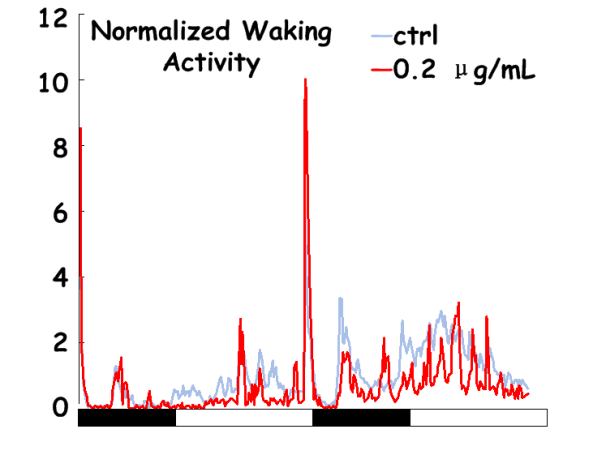

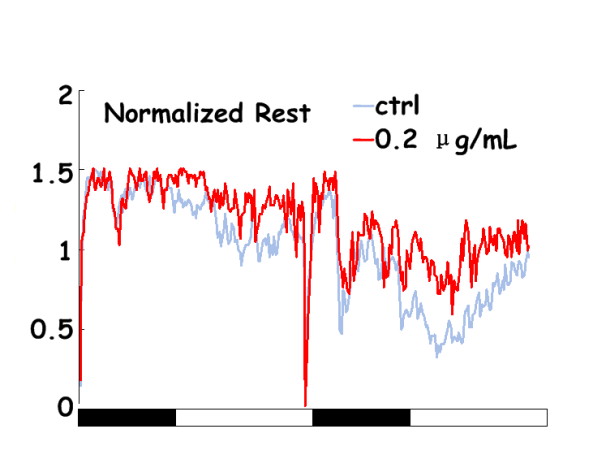


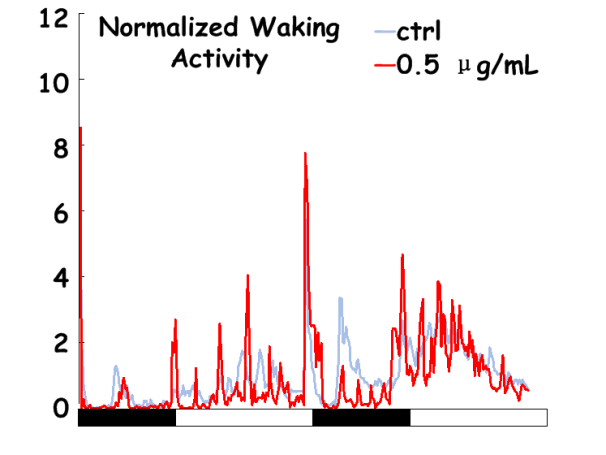

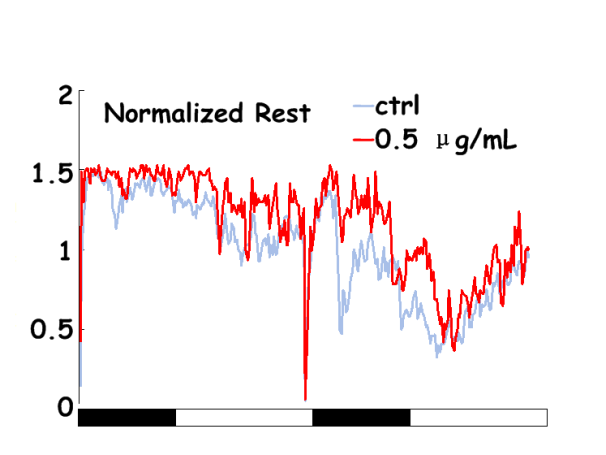

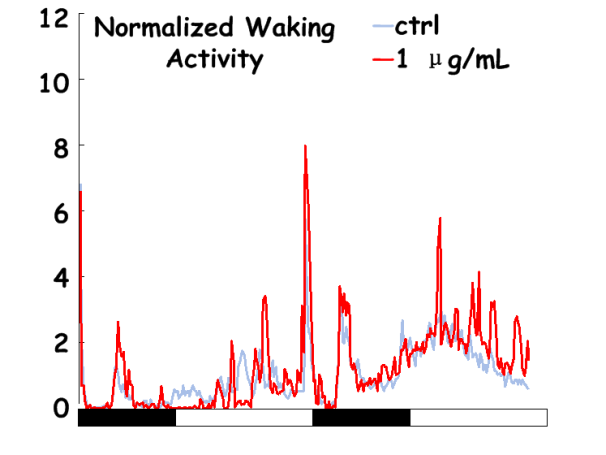

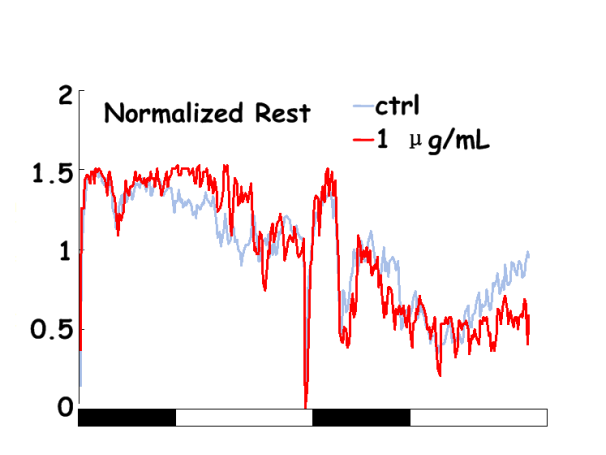


1227


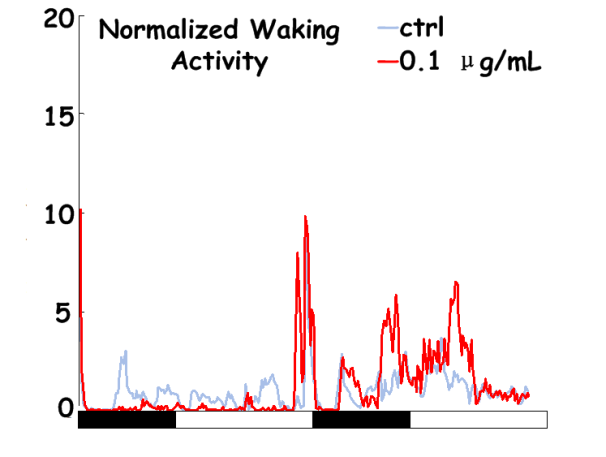

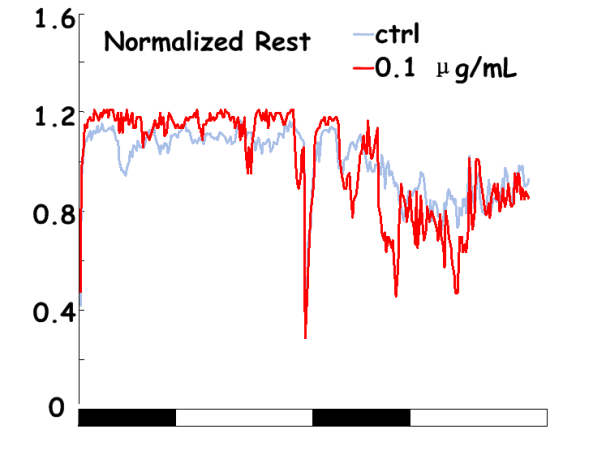

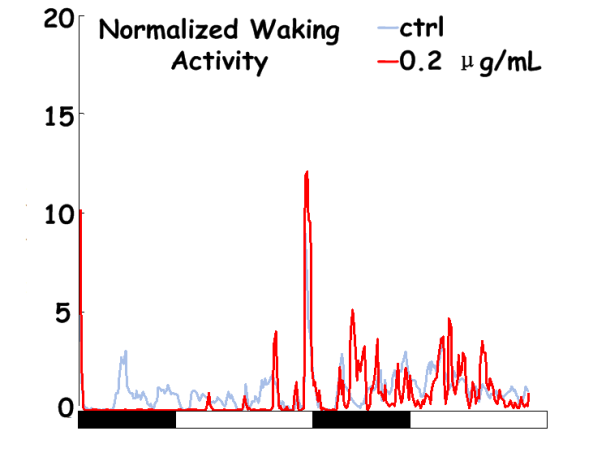

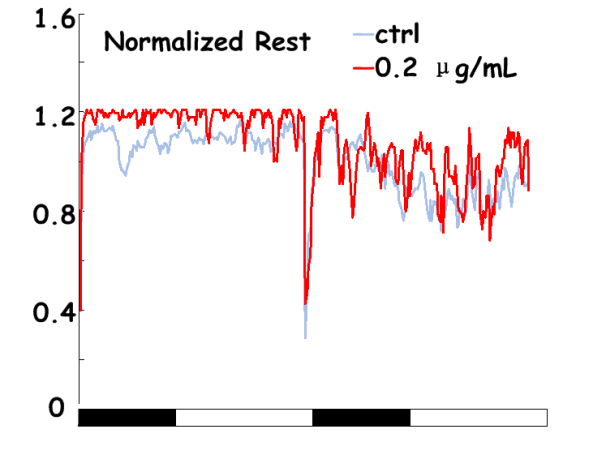

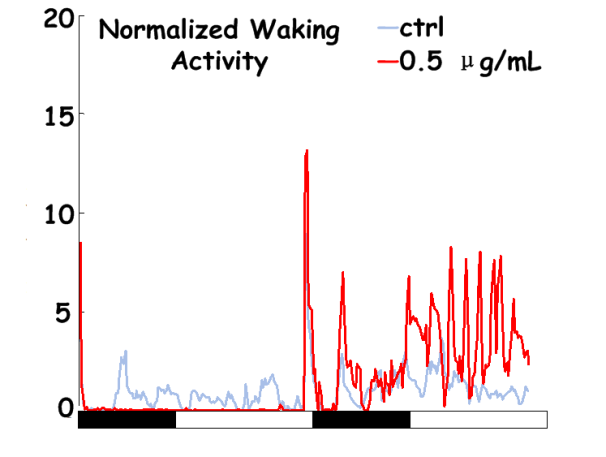

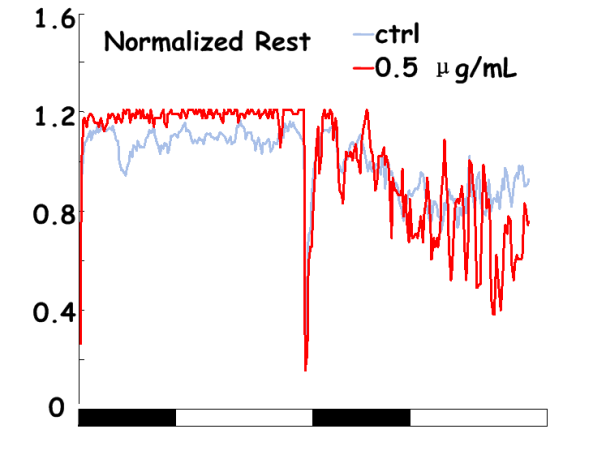

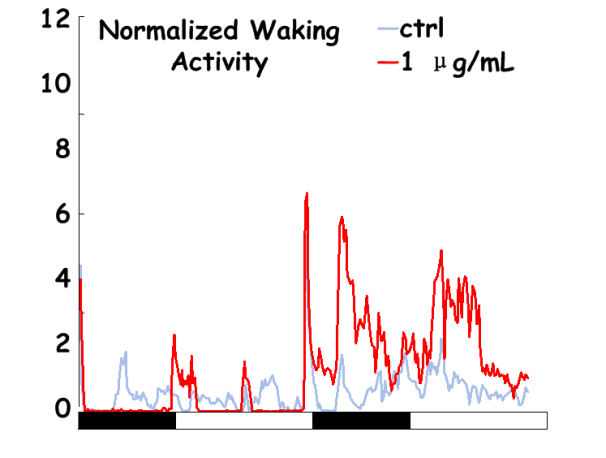

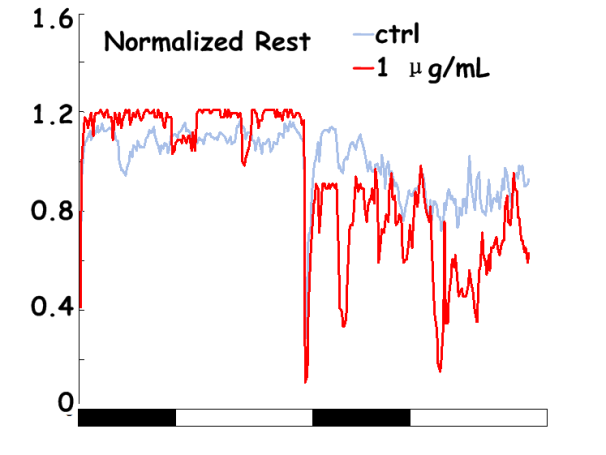


AEO


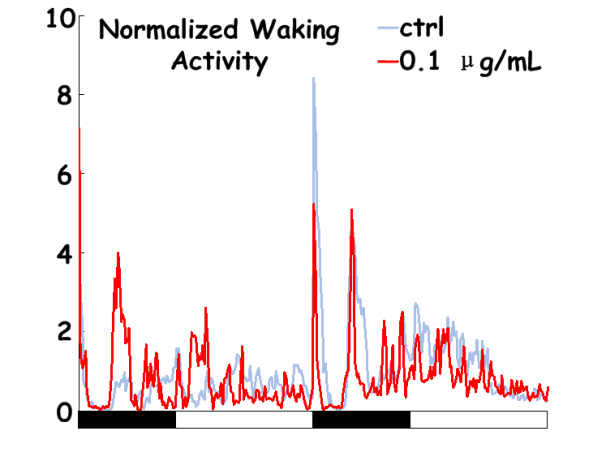

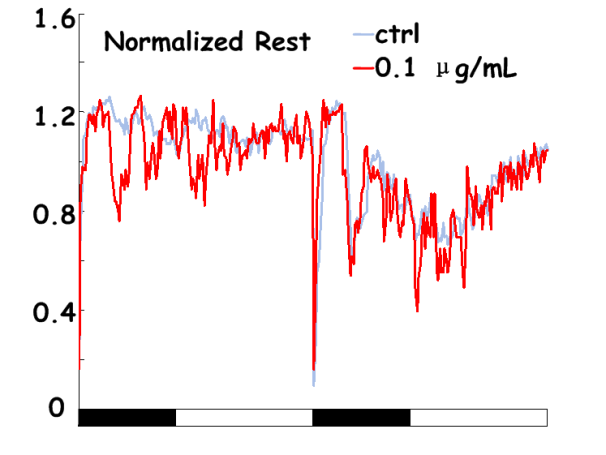

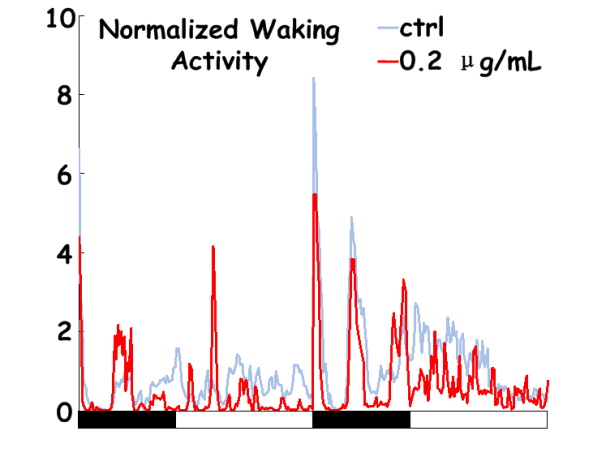

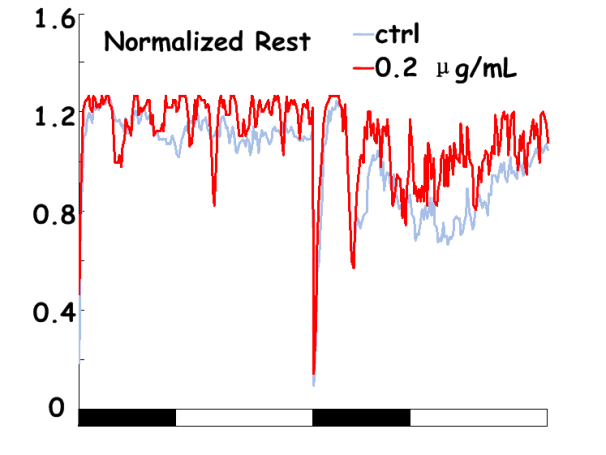

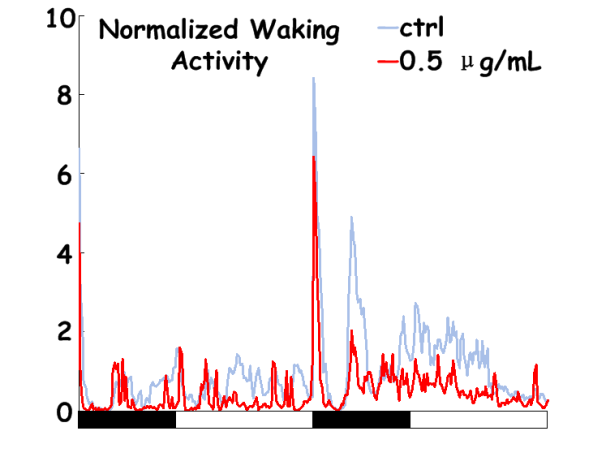

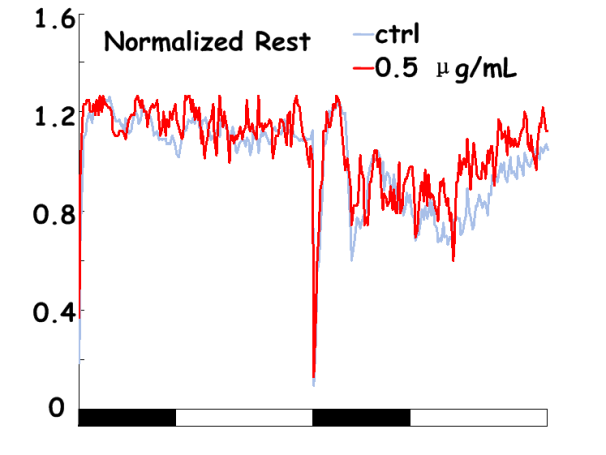

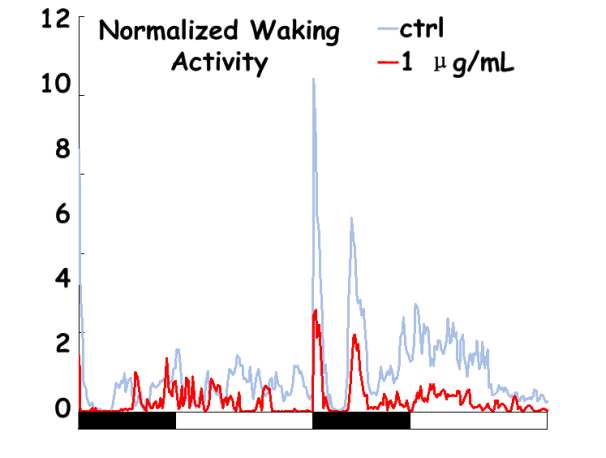

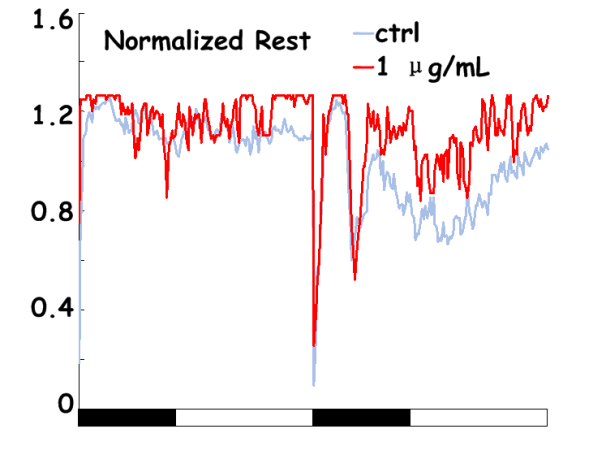


**Figure S2.** Rest total and waking activity were averaged in 10-minute intervals and then normalised from control values. The two parameters were used to plot the time series. The red and blue traces indicated the exposed group and control group. The black bars represented the night measurements and the white bars represented the day measurements.

**Figure S3**

SDS


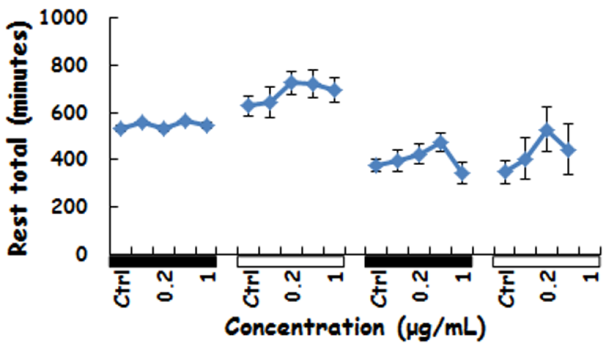

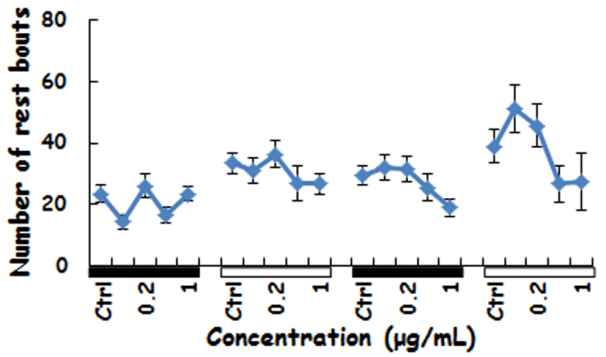

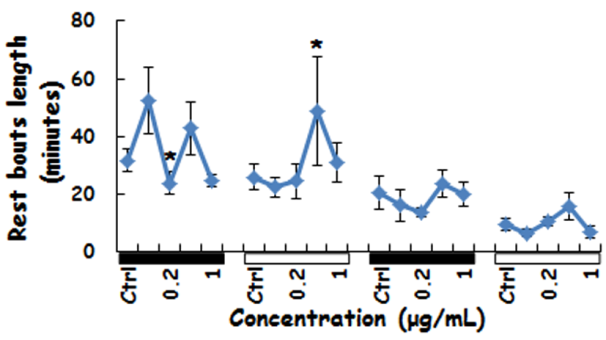

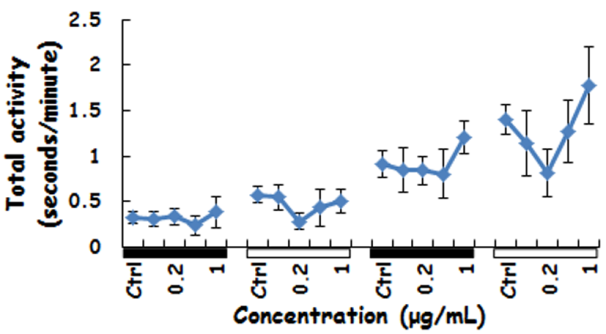

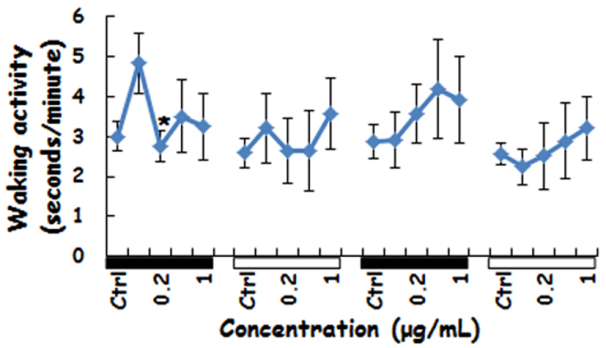


1227


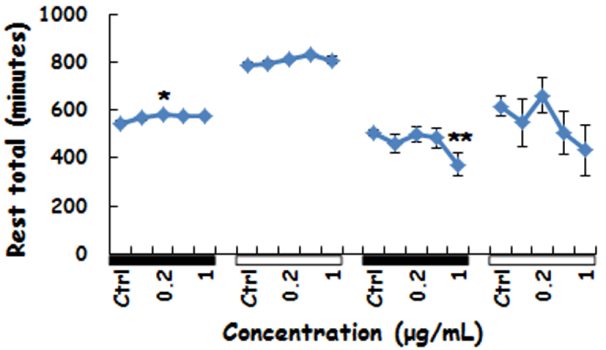

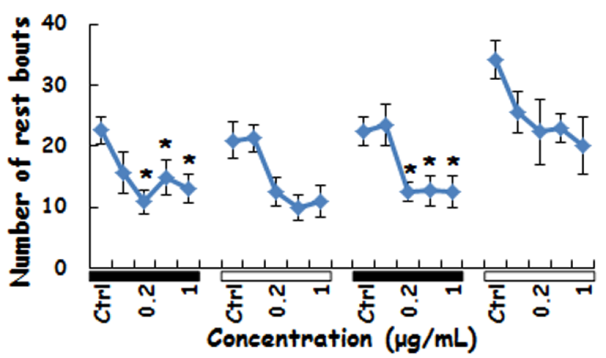

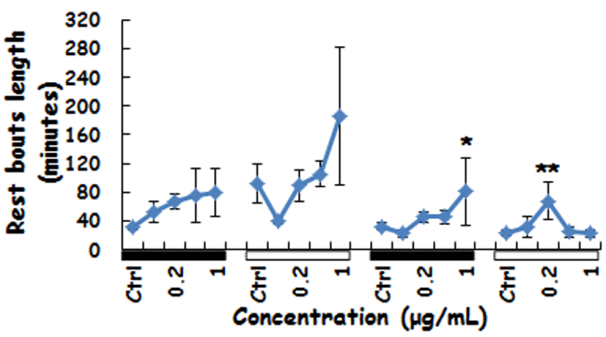

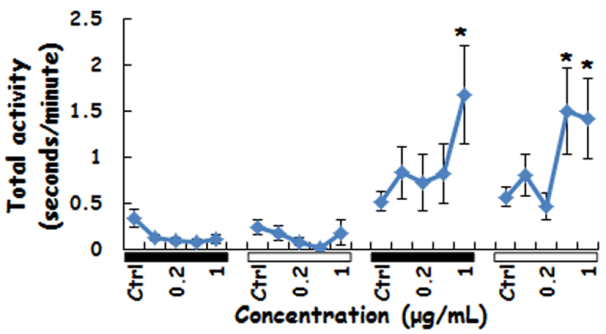

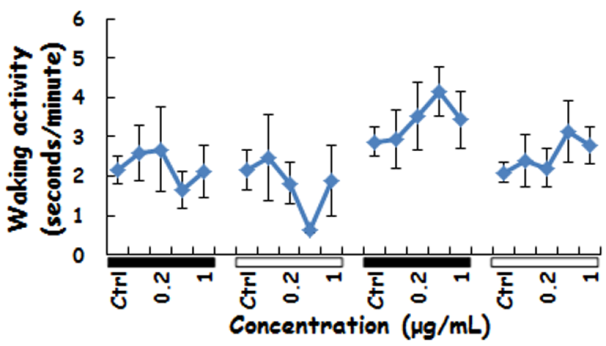


AEO


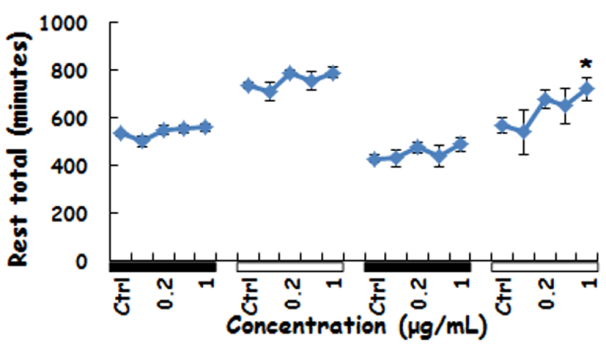

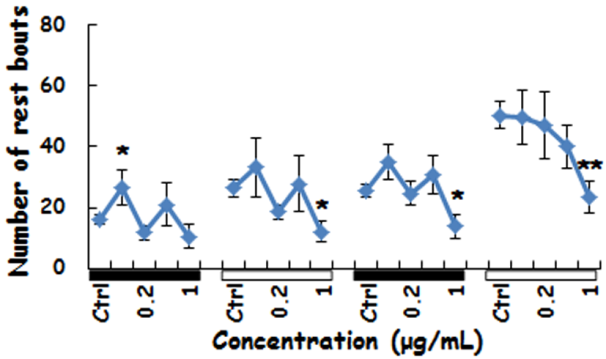

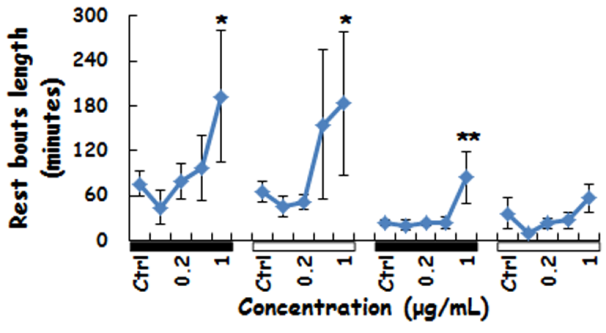

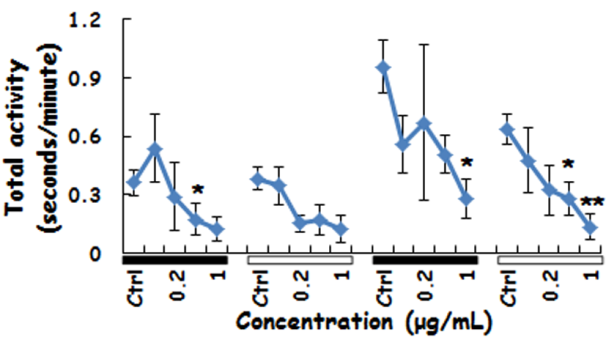

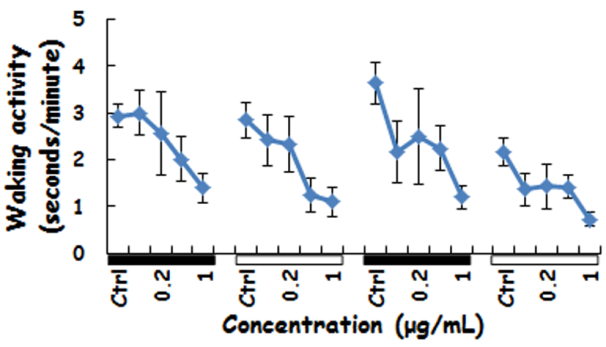


**Figure S3**. Statistical analyses of rest total, number of rest bouts, rest bouts length, total activity and waking activity. Each value was the average of ~48 larvae. The error bar represented the standard error of the means (SEM). The statistic significances were set at P < 0.05 (*) and P < 0.01 (**). The black bars indicated the night measurements, and the white bars indicated the day measurements.

**Figure S4** SDS


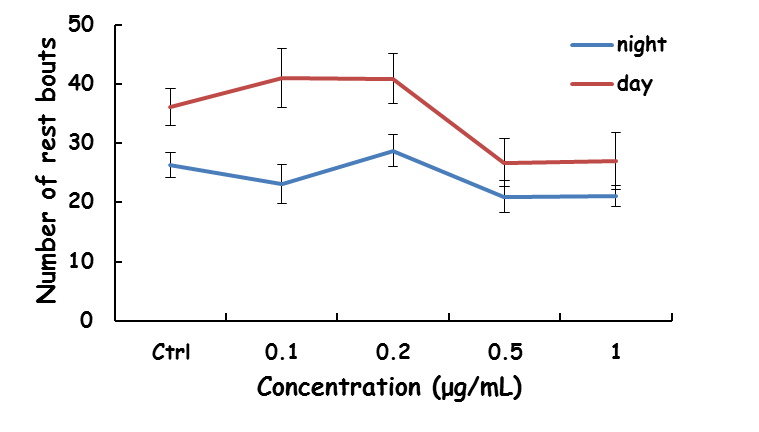

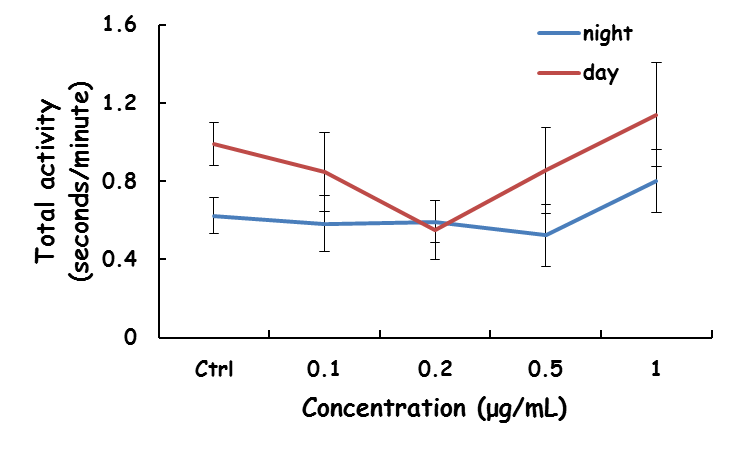


1227


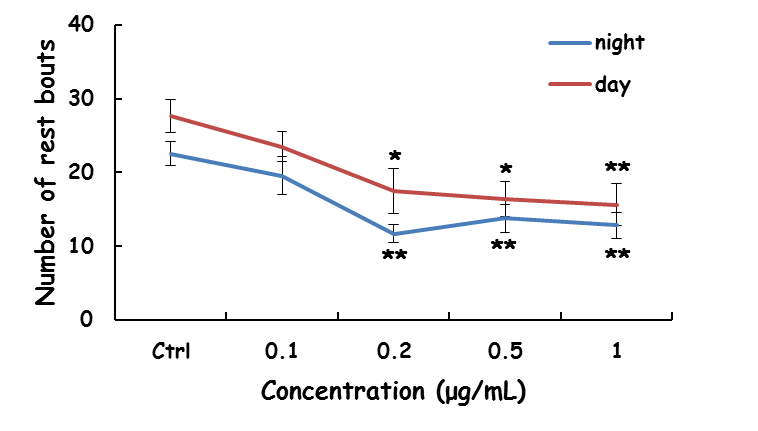

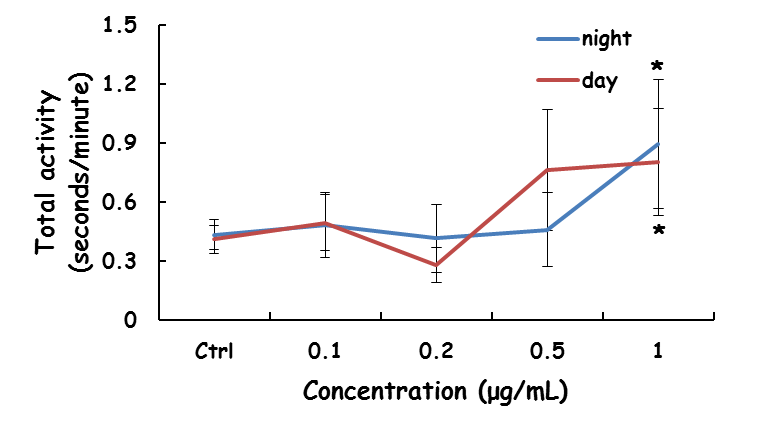


AEO


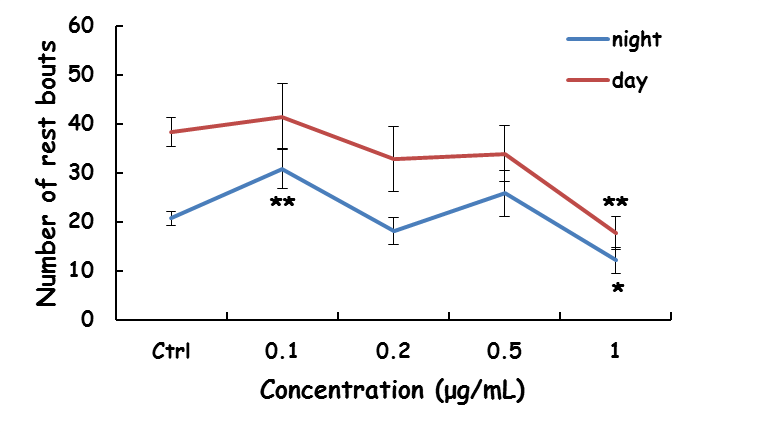

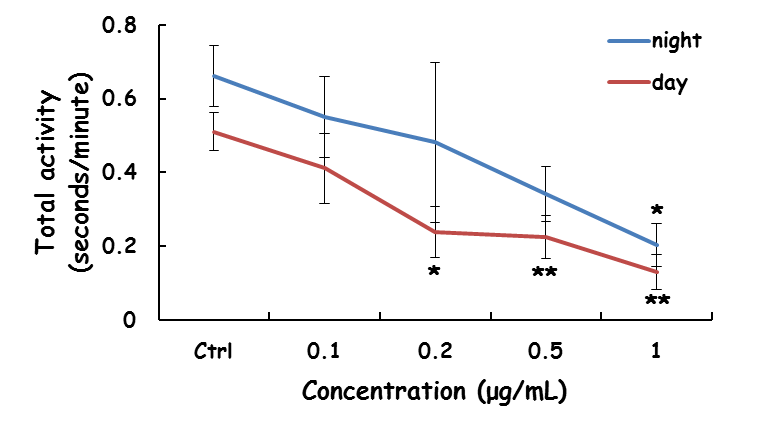


**Figure S4.** Statistical analyses of number of rest bouts and total activity during the day and night. The error bar represented the standard error of the means (SEM). The statistic significances were set at P < 0.05 (*) and P < 0.01 (**).

**Figure S5**


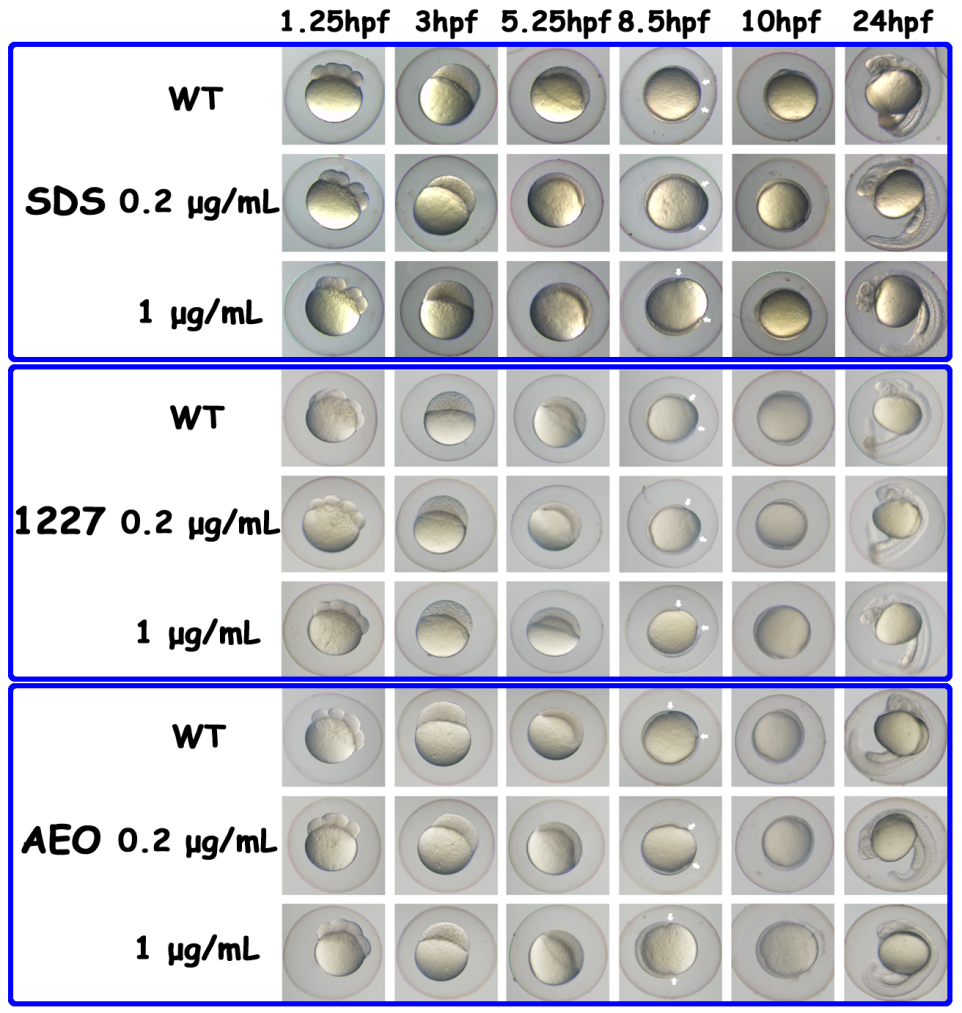


**Figure S5.** Morphological characteristics of embryos treated with three types of surfactants with different charges from 1.25 to 24 hpf. The arrows represented the start and end of epiboly.

**Figure S6**

**A**


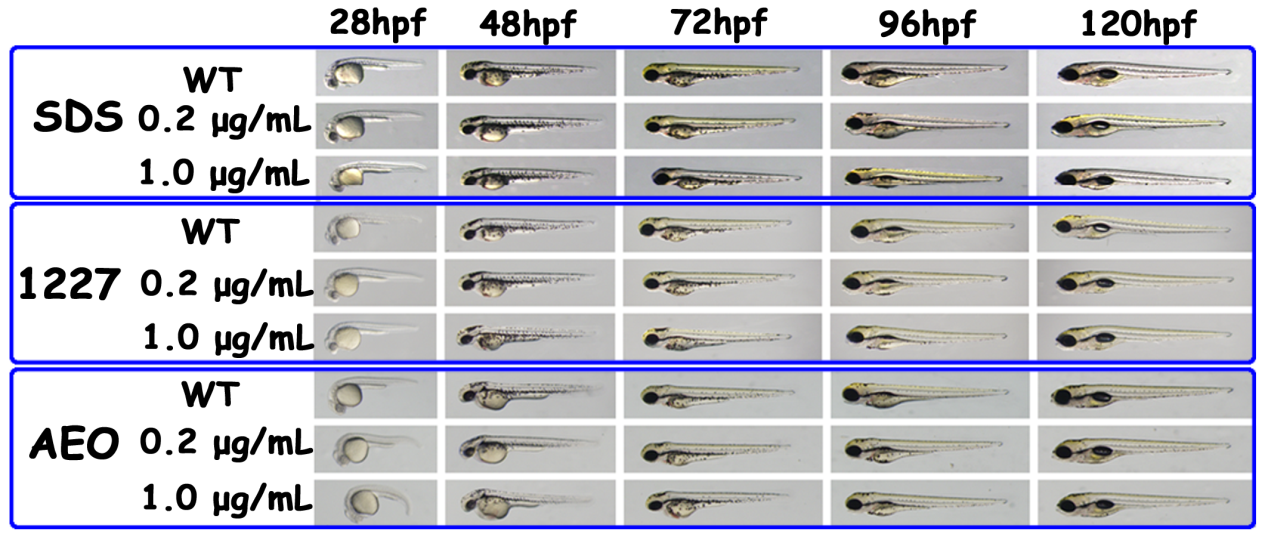


**B**

**C**

**D**

**Figure S6.** Effects of surfactants treatment on early development of zebrafish embryos from 28 to 120 hpf. **(A)** Morphological characteristics of embryos and larvae treated with three types of surfactants. **(B)** Statistical analyses for the body length of the treated embryos and wide type. **(C)** Statistical analyses for the eye area of the treated embryos and wide type. **(D)** Statistical analyses for the head area of the treated embryos and wide type. The statistical significances were set to P < 0.05 (*) and P < 0.01 (**)

**Figure S7**


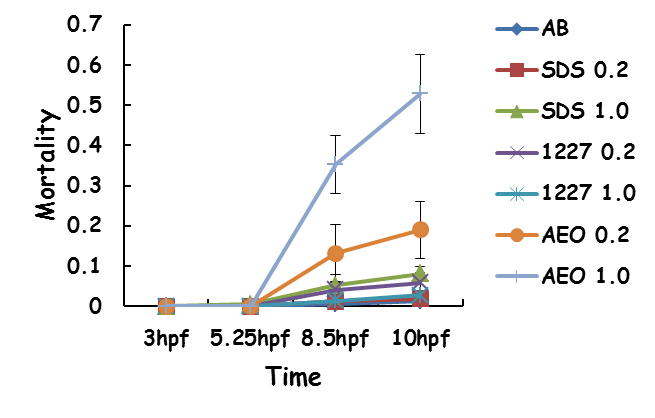


**Figure S7.** Mortality of zebrafish embryos treated with surfactants from 1.25 to 10 hpf. The error bar represents the standard error of the means (SEM).

**Figure S8**

**A**


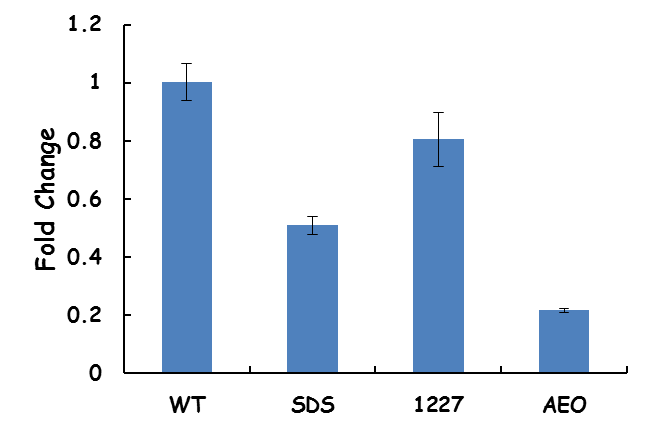


B


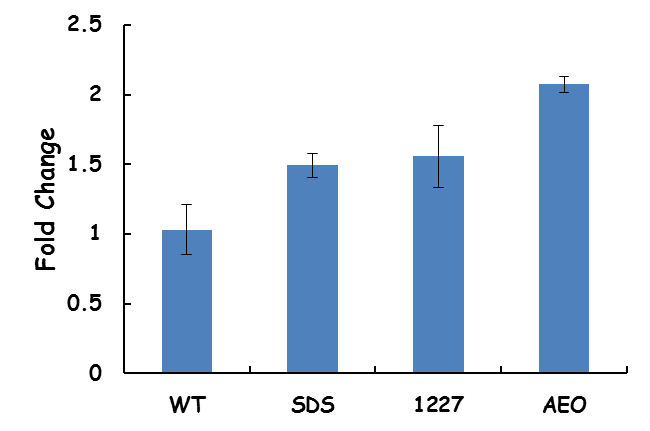


**Figure S8.** The expressions of *krox20* (A) and *ntl* (B) by qRT-PCR in zebrafish larvae.
